# A competition framework for fixation-preference in strabismus

**DOI:** 10.3389/fnins.2023.1266387

**Published:** 2023-10-18

**Authors:** Santoshi Ramachandran, Vallabh E. Das

**Affiliations:** College of Optometry, University of Houston, Houston, TX, United States

**Keywords:** strabismus, localization, superior colliculus, suppression, saccade, exotropia, nonhuman primates

## Abstract

Strabismic subjects often develop the ability to fixate on a target with either eye. Previous studies have shown that fixation-preference behavior varies systematically depending on spatial location of the target. We hypothesized that, when an eccentric target is presented, oculomotor fixation-preference in strabismus may be accounted for in a competitive decision framework wherein the brain must choose between two possible retinal errors to prepare a conjugate saccade that results in one of the eyes acquiring the eccentric target. We tested this framework by recording from visuo-motor neurons in the superior colliculus (SC) of two strabismic rhesus macaque monkeys as they performed a delayed saccade task under binocular viewing conditions. In one experiment, visual targets were presented at one of two locations corresponding to the neuronal receptive field location with respect to either the viewing or the deviated eye. Robust visual sensory responses were observed when targets were presented at either location indicating the presence of competing sensory signals for eye-choice. In a second experiment, a single visual target was placed at the neuronal receptive field location where the animal switched fixation on some trials and did not on other trials. At such target locations where either eye could acquire the target, both visual and build-up activity was greater in trials when the saccade encoded by the neuron “won.” These findings provide evidence for the influence of visual suppression within SC sensory activity and support the possible utilization of a competition framework, one that has been previously described for when a binocularly aligned animal chooses from among multiple targets, to drive fixation-preference behavior in strabismus.

## 1. Introduction

In our natural environment, we make frequent saccadic eye movements to foveate on objects of interest. With normal binocular vision, both eyes are aligned and coordinated when shifting gaze from one object to another. If binocular visual input is interrupted during development, disorders such as strabismus (ocular misalignment—fovea of each eye pointing in different directions) and amblyopia (reduced visual acuity in one or both eyes) can occur. Strabismus is a developmental disorder affecting up to 5% of children, worldwide, making it a significant public health issue (Mohney, 2007; Das, 2016; Walton et al., 2017).

In the presence of unilateral amblyopia, strabismic subjects will fixate and acquire targets with their “better-acuity” eye (fixating eye), and the fellow “lower-acuity” deviated eye will simply follow along. However, subjects with strabismus and minimal amblyopia can acquire and fixate eccentric targets with either eye (Steinbach, 1981; Sireteanu, 1982; Dickey et al., 1991). When the resulting saccadic eye movement leads to a change in the eye that is fixating, it is called fixation-switch behavior or alternating-saccade behavior (van Leeuwen et al., 2001; Das, 2009; Agaoglu et al., 2014). If the eccentric targets are visual, cortical suppression of information from parts of the retina, necessary to prevent double vision and visual confusion, influences fixation-preference or eye-choice behavior, and therefore this property of strabismus has led to significant insight into mechanisms of visual suppression. Thus, when tested with visual targets, fixation-preference varies systematically depending on the location of the target and the maps of spatial patterns of oculomotor fixation-preference show a close association with psychophysical measures of visual suppression (Sireteanu, 1982; Pratt-Johnson and Tillson, 1984; Von Noorden and Campos, 2002; Economides et al., 2012, 2014; Agaoglu et al., 2014). For example, in non-human primates with exotropia, targets to the left of the left eye were always acquired by the left eye and targets right of the right eye were always acquired by the right eye, fundamentally consistent with suppression of visual information from far temporal retina of each eye. The border between the right-eye and left-eye fixation zones developed at horizontal target locations that were approximately halfway between the lines of sight of the foveating and strabismic eyes, this time consistent with lack of suppression of temporal retina immediately adjacent to the fovea. This so-called border between right eye and left eye fixation zones is not sharply defined and the monkeys could acquire targets at these locations with either eye (Agaoglu et al., 2014). Interestingly, some recent work, including from our own lab, has shown that fixation-switch behavior can be elicited even in the absence of visual targets suggesting that some aspects are perhaps non-visual (Economides et al., 2014, Ramachandran and Das, 2020). In the current study, we attempted to investigate the neural basis for fixation-preference by performing neurophysiological recordings in the superior colliculus (SC) of strabismic non-human primates. The SC was chosen as the target of investigation due to its role in saccadic eye movements and gaze reorientation to multimodal stimuli, target selection from among multiple targets, and as part of a decision network for saccadic eye movements (Jay and Sparks, 1984; Munoz and Wurtz, 1995).

The SC has been studied previously for target choice in normal non-human primates (McPeek and Keller, 2002; Li and Basso, 2005; Kim and Basso, 2008) and it is evident that the SC, along with the frontal eye fields (FEF) and lateral intraparietal area (LIP), constitutes a target selection circuit and that the SC as a whole represents a gateway for target selection signals to be converted into a saccadic command (McPeek and Keller, 2002). Why might a target selection framework be relevant for eye-choice behavior in strabismus? In strabismus, because the eyes are not aligned, a single target could theoretically produce two retinal error signals leading to a scenario where the brain must choose between two possible saccade vectors that would bring one or the other eye as the fixating eye at the end of the saccade, the other eye assuming its label as the deviated eye. Indeed, using a sparse noise stimulus during single-cell recording of SC visual neurons, it has been reported that the receptive fields, of the fixating and deviated eye, are simply offset in position by the magnitude of ocular deviation (Economides et al., 2018). The goal of this study was to investigate the neural substrate for fixation-preference behavior in strabismus and determine if a competition-framework analogous to target selection in a normally aligned subject could serve as the mechanism that the brain uses to elicit fixation-preference behavior in strabismus. Some of these results have been previously published in abstract form (Ramachandran and Das, 2022).

## 2. Methods

### 2.1. Subjects, animal model, and surgical procedures

The subjects of this study were two adult (one male, one female) exotropic (XT-divergent strabismus) macaque monkeys (*Macaca mulatta:* M1: ~30°XT and M2: ~30–35° XT; vertical deviation of ~5° in M1 and ~ 1° in M2), whose strabismus was previously induced in infancy by disrupting binocular vision during the critical period of development using an optical prism-rearing method. In the optical prism-rearing paradigm, the infant monkeys wore lightweight helmets fitted with either a base-in or base-out prism in front of one eye and a base-up or base-down prism in front of the other eye starting from day 1 after birth till they were 4 months of age after which they were allowed to grow under unrestricted viewing conditions (Smith et al., 1979; Crawford and von Noorden, 1980). After birth, animals undergo a routine clinical exam to ensure that they meet normal standards of health to be included in the study. During the rearing period, the helmets are modified periodically to account for the changing head size of the animal during the rearing period. This rearing paradigm decorrelates binocular vision during the critical period for visual development thus resulting in development of strabismus and many other visual and oculomotor properties of strabismus including fixation-preference behavior (Tusa et al., 2002; Das, 2016).

When the animals were ∼4 years of age, they underwent a surgical procedure carried out under aseptic conditions with isoflurane anesthesia (1.25–2.5%) to implant a head stabilization post (Adams et al., 2007). Later in a second surgery, we stereotaxically implanted a 21-mm diameter titanium recording chamber targeting the SC in each animal. In the same surgery, we also implanted a scleral search coil in one eye using the technique of Judge et al. (1980) and in a third surgery, a scleral search coil was implanted in the fellow eye. All procedures were performed per National Institutes of Health guidelines and the ARVO Statement for the Use of Animals in Ophthalmic and Vision Research and the protocols were reviewed and approved by the Institutional Animal Care and Use Committee (IACUC) at the University of Houston. Monkey M1 was used in our previously published study (M1) that examined the behavioral aspect of spatial patterns of fixation preference in strabismic monkeys when presented with auditory vs. visual stimuli (Ramachandran and Das, 2020).

### 2.2. Experimental paradigm, data acquisition, and statistical analysis

During testing, the head-fixed monkey was seated 57 cm away from an array of visual targets made up of 21 red light-emitting diodes (LEDs) that were ~ 1° in diameter. The 21 LEDs, all spaced 10° apart, were arranged in three horizontal rows of seven LEDs (each row separated vertically), resulting in seven possible horizontal location components (−30°, −20°, −10°, 0°, +10°, +20°, +30°) and three possible vertical location components (−10°, 0 and +10°). The visual stimuli were controlled by custom programs developed using LabVIEW (National Instruments, Austin, TX, United States). The eye coil signal was calibrated at the beginning of each experiment as the animal monocularly fixated the series of LEDs along the horizontal (−30° to 30°) and vertical (−10° to 10°) meridians. The animal was rewarded with small amounts of juice when viewing within a small region (±2°) around the target.

**Figure 1:**
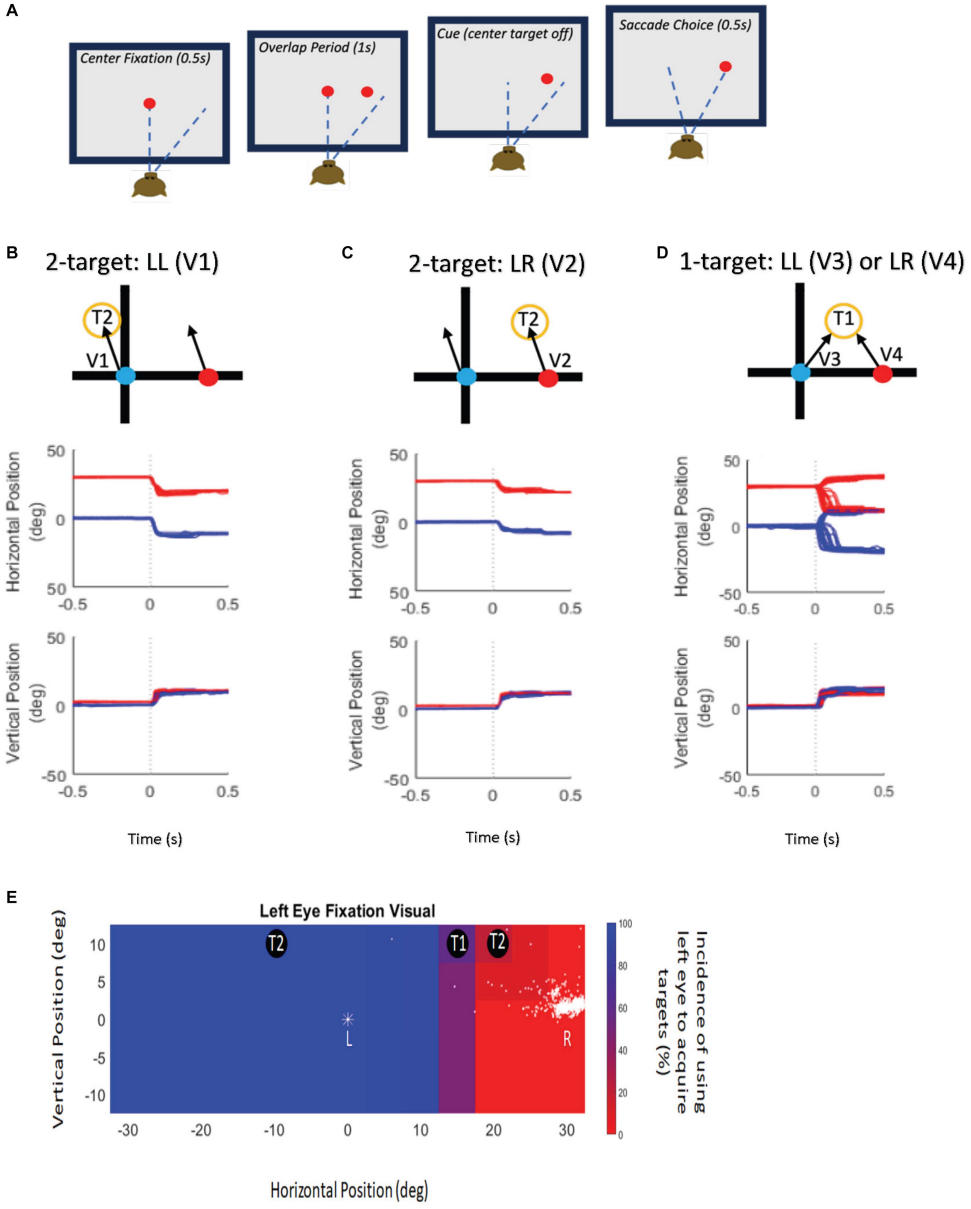
Experimental paradigm and target presentation. **(A)** Schematic display of the delayed saccade task. Eccentric target was presented while the fixation stimulus was present with an overlap of 1 s (second panel—sensory period). Reward reinforcement was contingent upon delaying saccade initiation until the fixation stimulus disappeared (third panel), after which the monkey made a saccade to the target (fourth panel—motor period). **(B)** *Top panel*—Schematic representation of target locations and possible saccade vector (V1) during twotarget testing. Schematic shows the left eye (blue symbol) initially fixating and the right eye (red symbol) is deviated and non-fixating. The fixating, left eye obtains the target. Examples of raw data trials of the schematic representations (V1) shown in the *Middle* (horizontal eye position) and *Bottom* panels (vertical eye position) illustrate the behavioral outcomes of two-target testing when the fixating eye obtains the target located at (−10, 10) on 100% of the trials (V1). **(C)** *Top panel*—Schematic representation of target locations and possible saccade vector (V2) during two-target testing. Schematic shows the left eye (blue symbol) initially fixating and the right eye (red symbol) is deviated and non-fixating. The deviated, right eye obtains the target. Examples of raw data trials of the schematic representations (V2) shown in the *Middle* (horizontal eye position) and *Bottom* panels (vertical eye position) illustrate the behavioral outcomes of two-target testing when the initially deviated eye obtains target located at (20, 10) on 100% of the trials (V2). **(D)** *Top panel*—Schematic representation of target locations and possible saccade vectors (V3, V4) during one-target testing. Schematic shows the left eye (blue symbol) initially fixating and the right eye (red symbol) is deviated and non-fixating. T1 locations resulted in the monkey using either eye to acquire the target that may change on a trial-to-trial basis. Examples of raw data trials of the schematic representations (V3-V4) shown in the *Middle* (horizontal eye position) and *Bottom* panels (vertical eye position) illustrate V3 and V4 in one-target testing when the initially fixating left eye obtains the target located at (10, 10) on some trials and when the initially deviated right eye acquires the target on other trials. **(E)** Target presentation schematic illustrates example locations for one-target (T1) and two-target (T2) testing overlaid on the behavioral spatial patterns of fixation-preference for monkey M1. The pseudo-color plot shows eye choice behavior for trials where the left eye was initially fixating the central target. The position of the initially fixating eye is indicated by a “*” symbol. The position of the deviated (non-fixating) eye is shown as dots and is variable from trial to trial. Eccentric target locations where the left eye acquired the eccentric target on 100% of trials are shown in blue, and target locations where the right eye was used to acquire the target on 100% of trials are shown in red. Intermediate percentages of eye choice are represented by the color scale shown next to the plot. Therefore, at T2 locations, either the left eye acquired the target on 100% of the trials (blue region) or the right eye acquired the target on 100% of the trials (red regions) and are used for two-target testing. T1 locations are those where either eye could acquire the target and are used for one-target testing. Note that the T1 and T2 locations also depend on the visual receptive field location of the neuron that is being recorded.

Eye position data from both eyes were collected as each animal performed a classical delayed saccade task under binocular viewing conditions (Figure 1). In delayed saccade testing, the monkeys were presented with a central fixation target (Figure 1A—baseline) for 500 ms after which an eccentric target was turned on for 1,000 ms (Figure 1A—sensory overlap period) during which they continued to maintain central fixation. Once the central fixation LED was extinguished, the monkeys made a saccade to the eccentric target location within 500 ms (Figure 1A—motor period). The fixation/saccade reward window was 5° but this was largely unnecessary since the saccades were made to a visual target and were therefore very accurate. The eccentric target was presented at a specific horizontal and vertical location corresponding to the retinotopic receptive field location of the cell with respect to either the fixating or the deviated eye. If the preferred amplitude of the neuron was larger than 10–15° in the vertical direction, the initial fixation LED was displaced vertically to either the −10° or + 10° location because we were limited in the range of vertical target locations. We did not need to offset the horizontal starting position because the available LED array had a wide horizontal range (±30°). Since all testing was performed under binocular viewing, the strabismic monkeys in this study were free to choose either eye for central fixation and also free to acquire the eccentric target with either eye leading to trials of fixating eye acquiring the target (no-switch or non-alternating saccade) or deviating eye acquiring the target (fixation-switch or alternating saccade) that were sorted and analyzed separately.

Initial exploration and evaluation of the SC neural responses was qualitative prior to acquiring data from isolated units for later quantitative analysis. Thus the SC was identified by visual only responses from cells in the superficial layer upon presentation of the eccentric target in the delayed saccade paradigm followed by cells with visual and saccade related bursting as we descended into the intermediate and deep layers. Once the approximate receptive field location of the isolated SC visuomotor neuron was identified, the optimal location of the receptive field was determined by identifying the LED target location, from among the available adjacent LED targets that provided the most robust firing response. The same process was done separately for the viewing eye and the deviated eye to map the receptive field locations for each eye independently. When possible, electrical stimulation, resulting in staircase saccades, was also used to map the area. Once a visuo-motor cell was isolated and its receptive field (preferred LED location) was identified, the following testing paradigms (Figures 1B–D) were carried out under binocular viewing conditions.

- Two-Target Testing: If the neuronal receptive field location fell on areas where the animal either always switched fixation or never switched fixation (Figures 1B,C,E), we performed two-target testing. In two-target testing, visual targets were placed at one of two possible locations (T2 in Figures 1B,C)—the receptive field location corresponding to the fixating eye or that corresponding to the deviating eye, resulting in either a V1 or V2 saccade (Figures 1B,C). The goal of this testing was to investigate if the neuron encodes visual information available from either the fixating or the deviated eye leading to a corresponding motor (saccade) output. Data analysis included comparing visual, build-up and motor responses of the SC neuron when a target is presented in the receptive field corresponding to the fixating or the deviated eye. A neural visual response at either receptive field location would indicate that visual information is available to both eyes. Example eye traces for two-target testing is shown in Figures 1B,C.
- One-Target Testing: For certain target locations (T1 in Figures 1D,E) that lie approximately midway between the gaze axes of the two eyes, the animals spontaneously change their eye of fixation on a subset of trials (Figure 1D). We performed one-target testing (Figure 1D) on cells whose neuronal receptive fields lay in these target areas. Such cells and trials were analyzed separately within the one-target testing paradigm because the neural responses presumably were reflective of a competition between two possible saccade vector choices (V3, V4) emanating from the left and right colliculi. The goal of this testing was to compare neuronal activity on trials when the saccade outcome was corresponding to the preferred direction for the neuron (no-switch trials; V3 saccades) to trials in which the saccade outcome was in the opposite direction and the previously deviated eye acquired the target (fixation-switch trials; V4 saccades). Example eye traces for one-target testing is shown in Figure 1D.

Since a discrete set of LEDs were used as targets, it is possible that the LED that was selected as the eccentric target was not located at the exact center of the SC neuronal receptive field of either the viewing or the deviated eyes. The effect of being slightly away from the center of the receptive field could be a small broadening of the SC motor response and slight depression of the peak visual and motor response (Munoz and Wurtz, 1995). However, this does not affect the comparisons because we were comparing across trials that were either saccade matched (two-target paradigm) or at a singular location (one-target paradigm).

Neural data were recorded using epoxy coated tungsten electrode with ∼1 Mohm resistance (Frederik Haer, Brunswick, ME, United States). Binocular eye and raw spike data were acquired at a sampling rate of 1,000 Hz and 30 kHz, respectively, within the Blackrock Cerebus system (Blackrock Neurotech, Salt Lake City, UT, United States). Spike sorting was performed offline using Blackrock Offline Spike Sorter (BOSS Software; Blackrock Neurotech, Salt Lake City, UT, United States). Unit response was represented as a spike density function that was generated by convolving action potential time stamps with a 15-ms Gaussian (Richmond et al., 1987). Further data analysis was performed with custom software routines developed in MATLAB (MathWorks, Natick, MA, United States), and SigmaPlot 12.0 (Systat, Inc.; San Jose, CA, United States) was used for statistical analysis. Neuronal activity for each cell was divided into a visual period, early and late build-up periods and motor period and for each period we compared peak firing rates when the fixating eye (fixating on the center target at the start of trial) obtained the eccentric target (no-switch) to when the deviated eye (non-fixating eye at the start of the trial) obtained the eccentric target (fixation-switch). The overall goal of the data analysis was to establish whether changes in neuronal firing rates observed in SC cells was predictive of eye-choice behavior (Ramachandran and Das, 2020).

## 3. Results

We recorded from 72 SC neurons, in two strabismic monkeys during a delayed saccade task, that showed the classical visuomotor behavior of an initial transient sensory response upon presentation of the eccentric visual target, buildup activity during the delay period and then a strong motor response correlated to the execution of the saccade to the eccentric target (Lee et al., 1988; Glimcher and Sparks, 1992; Munoz and Wurtz, 1995; Basso and Wurtz, 1998; Horwitz and Newsome, 2001; Gandhi and Katnani, 2011). For each neuron, we first estimated the approximate center (given the limitations of the discrete array of targets) of the visual and motor receptive fields by online identification of the eccentric target location and subsequent saccade direction and amplitude that yielded maximal activity. Thereafter delayed saccade testing began under binocular viewing conditions with either two-target testing or one-target testing.

### 3.1. Neural responses during fixation-switch and no-switch trials: two-target testing

**Figure 2:**
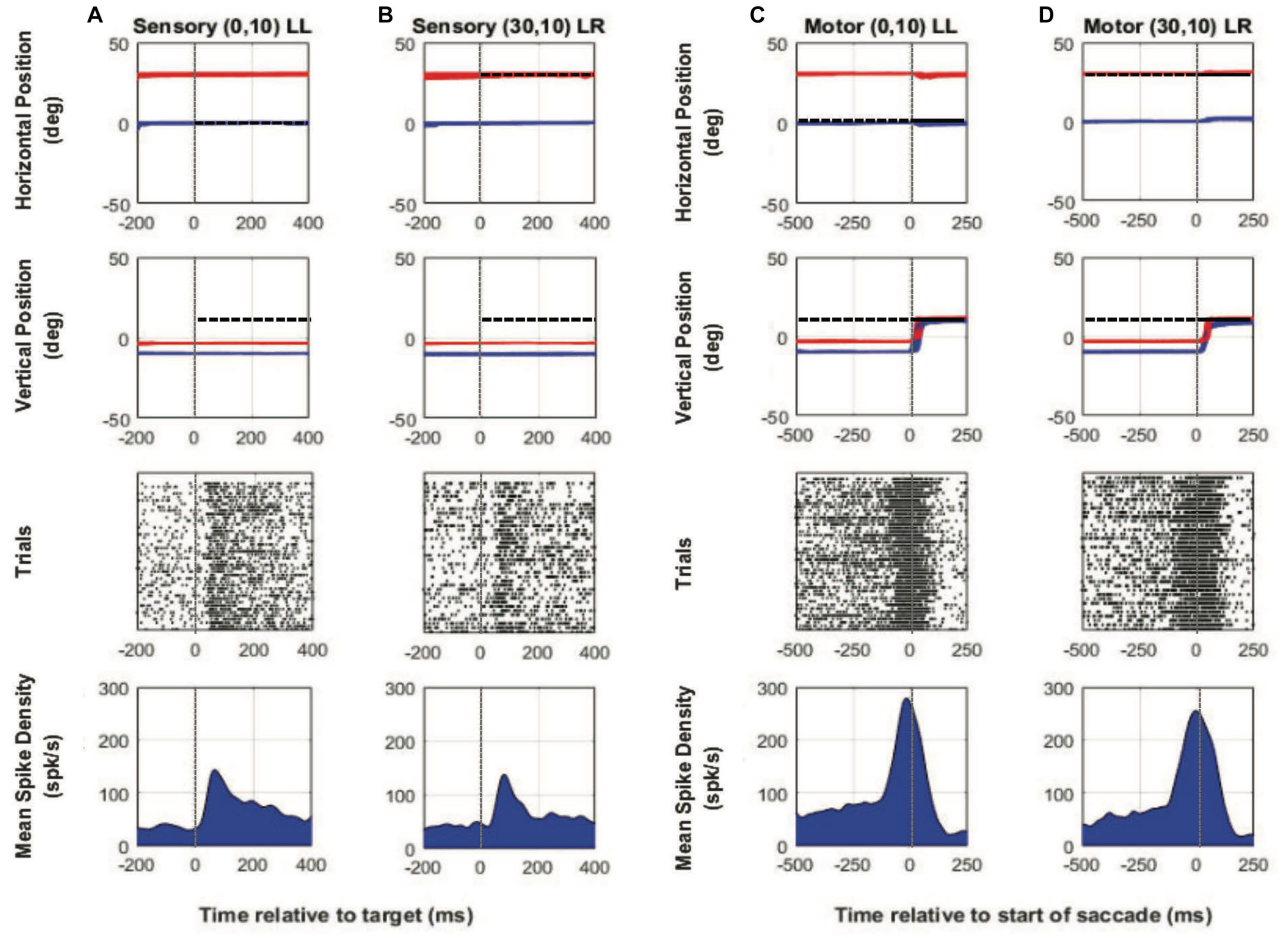
Two-target testing example cell (M1). Target-aligned eye and neural data (columns **A** and **B**) and saccade-aligned eye and neural data (columns **C** and **D**) when the monkey used either the initially fixating left eye (columns **A,C**—LL trials) to acquire the eccentric target or the initially deviated right eye to acquire the eccentric target (columns **B,D**—LR trials). In each column, top row is horizontal eye position (red—right eye, blue—left eye, and black—target), second row is vertical eye position, third row is raster showing incidence of neuronal spikes on each trial (each row in raster represents a single trial), and fourth row is the mean neuronal spike density function. Positive numbers on the position axes are rightward or upward positions and negative numbers are leftward or downward. For this example neuron, the preferred vector was close to a vertical 20° movement and therefore the initial fixation point was located at down 10°. For trials in which the fixating left eye acquired the target, the eccentric target was placed at (0° Hor, 10° Ver) which was the receptive field location appropriate for the left eye and for trials in which the deviated right eye acquired the target, the eccentric target was placed at (30° Hor, 10° Ver) which was the equivalent receptive field location appropriate for the right eye. Sensory neuronal responses in columns **(A)** and **(B)** show that placing the target at either location resulted in a robust visual response (mean peak spike density sensory—LL peak: 143.1 spks/s, LR peak: 137.1 spks/s). Motor responses when either eye acquires the target are also robust and similar because the eye vectors are similar (mean peak spike density motor—LL peak: 278.4 spks/s, LR peak: 254.5 spks/s).

Figures 2A–D show example neural and binocular eye data from a SC visuomotor cell in M1 (~30 deg. of exotropia) tested during the two-target paradigm with the left eye initially fixating the central target. Figures 2A,C show target-aligned and saccade-aligned data for when the target was placed at a location vertically up from central fixation (0,10), which was the receptive field location with respect to the fixating left eye and when the same eye, i.e., the initially viewing left eye, acquired the eccentric target (no-switch). Figures 2B,D show target-aligned and saccade-aligned data for when the target was placed at an equivalent vertically up location (30, 10) from the deviated right eye and when the deviated eye (right eye) acquired the eccentric target via a fixation-switch saccade. Note that in Figures 2C,D, the fellow eye makes a similar amplitude saccade and so saccade conjugacy is preserved; just the eye that is acquiring the target is changed. For each condition, only trials where saccade vector amplitudes were matched (t-test, p > 0.05) were selected for analysis. A primary observation was that visual responses (Figures 2A,B) are robust and fairly similar when the targets are presented at the equivalent receptive field locations with respect to the fixating or the deviated eye. Motor responses (Figures 2C,D) are also similar, which is to be expected since the saccade amplitudes and directions in the fixation-switch and no-switch conditions are matched.

**Figure 3:**
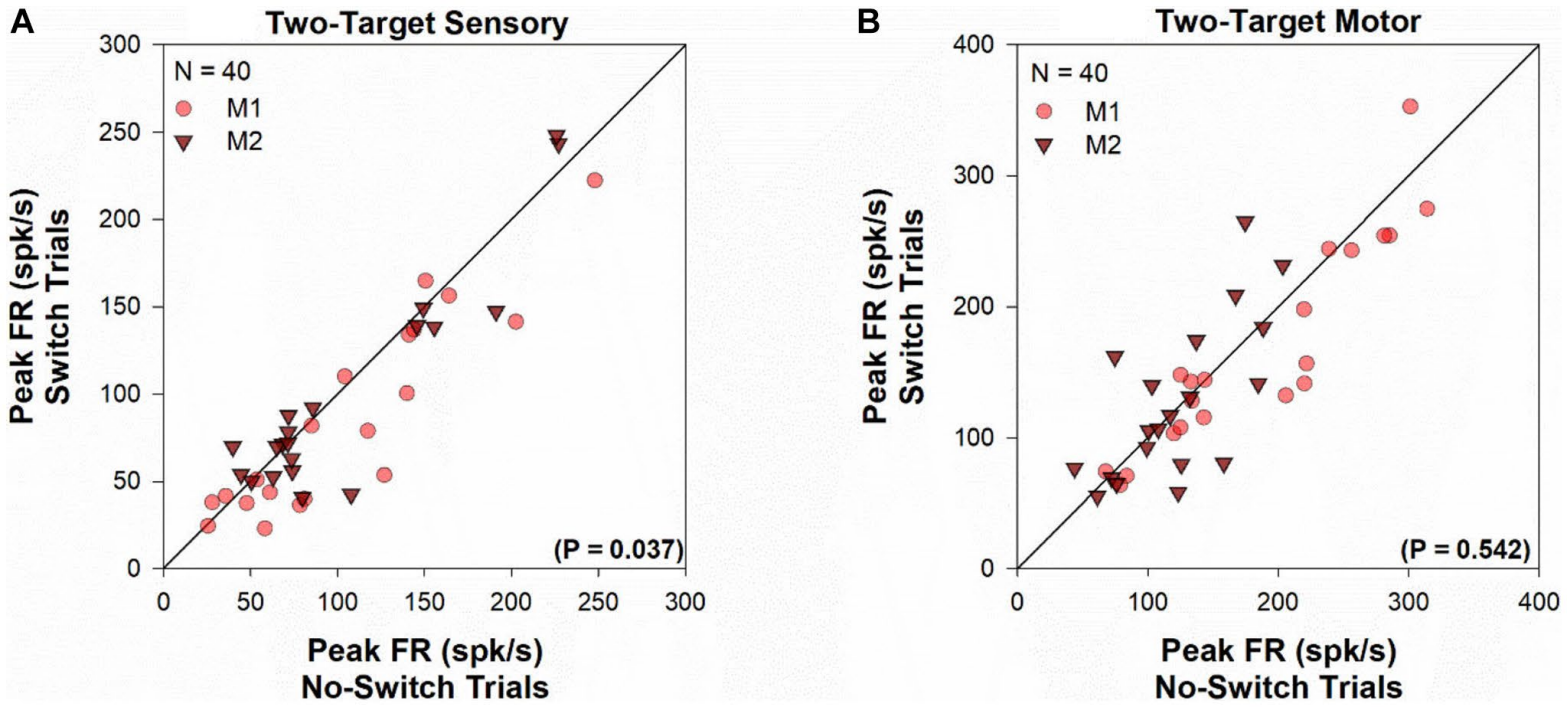
Two-target visual and motor peak population response. Comparison of the peak visual response (panel **A**) or peak motor response (panel **B**) between fixation-switch and no-switch trials of cell population during two-target testing. On the *x*-axis is the mean peak response for trials in which the initially fixating eye acquired the target (no-switch trials) and on the *y*-axis is the mean peak response on trials in which the initially deviated eye acquires the target (fixation-switch trials). Each symbol (circles—M1; triangles—M2) represents one cell. Peak visual responses were defined as the maximal value of the average instantaneous frequency response occurring within 100 ms of target onset. Peak motor responses were defined as the maximal value of the average instantaneous frequency response occurring within 100 ms surrounding saccade onset.

For each cell in the population, peak firing rates (Figure 3) were calculated for visual (Figure 3A) and motor (Figure 3B) periods, by finding the maximal value of the average instantaneous frequency response occurring within 100 ms of target onset and saccade onset, respectively. Every cell in our population showed significant visual response at the receptive field location for both the viewing eye and the deviated eye. However, peak firing rate for the sensory response was marginally greater when the viewing eye obtains the target (no-switch) vs. when the deviated eye obtained the target (fixation-switch; Z = -2.097, *p* = 0.037, Wilcoxon Signed Rank Test). This statistical observation suggests that although the receptive field location corresponding to the deviated eye is active, there may be small interocular suppression of the deviated eye by the viewing eye at the corresponding sites. As expected, for the two-target testing, peak motor responses for fixation-switch and no-switch trials were not significantly different since the saccade parameters were intentionally matched [*t*(39) = 0.616, *p* = 0.54, paired *t*-test]. It appears that although small interocular suppression may be present for sensory information applied to equivalent receptive field locations, it does not influence the final saccade that is generated.

**Figure 4:**
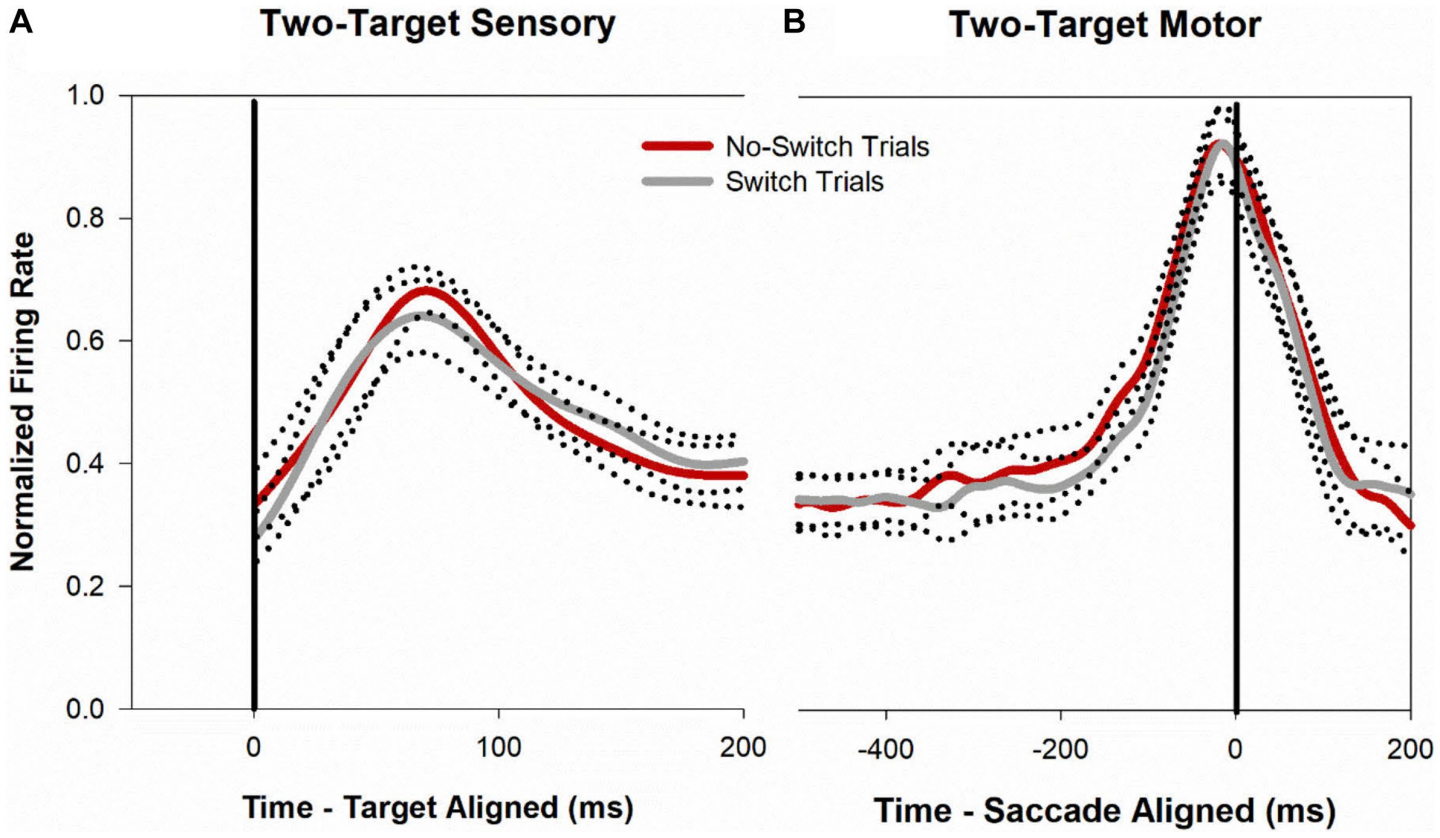
Normalized population (*n* = 40) activity of SC cells during two-target testing. Data are aligned at target onset (panel **A**; vertical line) or saccade onset (panel **B**; vertical line). Red trace is mean activity when the initially fixating eye acquired the target (no-switch trials) and gray trace is mean activity when the initially deviated eye acquired the target (fixation-switch trials). Dotted traces indicate ±standard error of the mean (SE). The neuronal response of each cell was normalized based on its maximal response and then a population mean was obtained by averaging the individual cell responses. Note that because each contributing neuron was normalized individually and did not necessarily reach a peak at the same time as other neurons, the mean population activity does not reach a peak value of 1.0.

**Figure 5:**
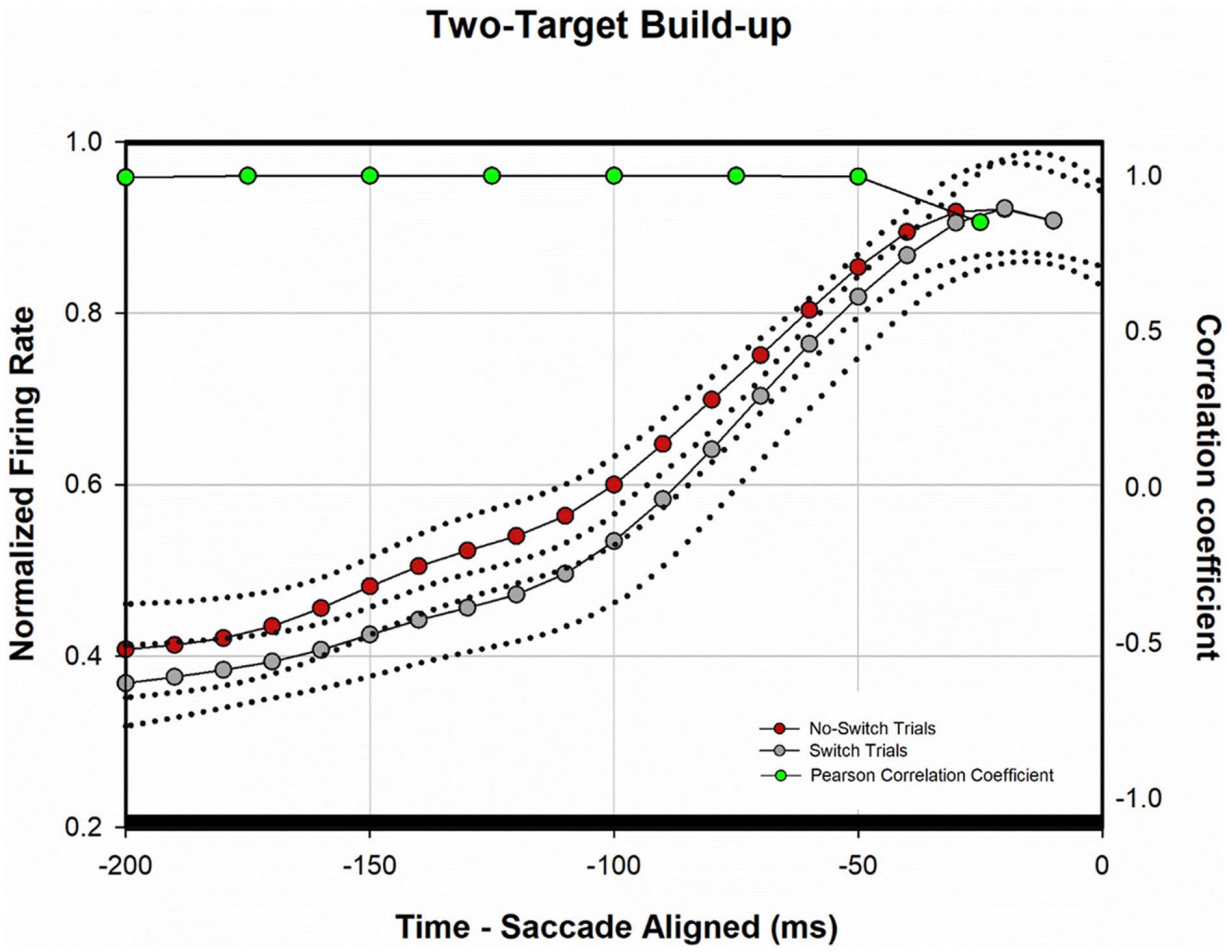
Analysis of normalized population activity during buildup period (*n* = 40). The traces show the mean population neural response during two-target testing for the 200 ms period prior to saccade onset (defined as time zero on the *x*-axis), which includes the buildup period. Dotted traces indicate ±SE. A Pearson correlation coefficient was developed with 25 ms bins of data between fixation-switch and no-switch trials. Black bars indicate a statistically significant positive correlation (Pearson Correlation) between fixation-switch (deviated eye acquires target) and no-switch (fixating eye acquires target) trials. The activity across the sample of buildup neurons during buildup period was similar if either eye acquired the target during two-target testing.

Figure 4 shows a temporal profile of the normalized discharge pattern of the population of neurons during the two-target paradigm. Once again, robust visual responses are present when the target is presented to the receptive location of the deviated eye (gray trace) or when the target is presented to the viewing eye (red trace). Peak motor response is almost identical for the two conditions. In order to analyze the buildup activity (activity just before the motor burst), we performed a correlation analysis similar to that described before (Mitchell et al., 2009). Briefly, from the normalized saccade aligned data (Figure 4B), Pearson correlations were developed between fixation-switch and no-switch trials during the 200 ms before saccade initiation. Data were divided into 25 ms bins to temporally identify statistical difference between neural responses when the fixating eye (no-switch trials) or the deviated eye (switch trials) acquired the target. Figure 5 illustrates the results of this analysis and shows that buildup activity (Figure 5) showed no difference in trials where the fixating eye or the deviated eye acquired the target and was positively correlated (i.e., firing rate increased toward motor burst regardless of which eye acquired the target). This finding is consistent with the fact that in both conditions the eventual saccade that acquires the target does so with similar amplitude and direction.

### 3.2. Neural response during fixation-switch and no-switch trials: one-target testing

**Figure 6:**
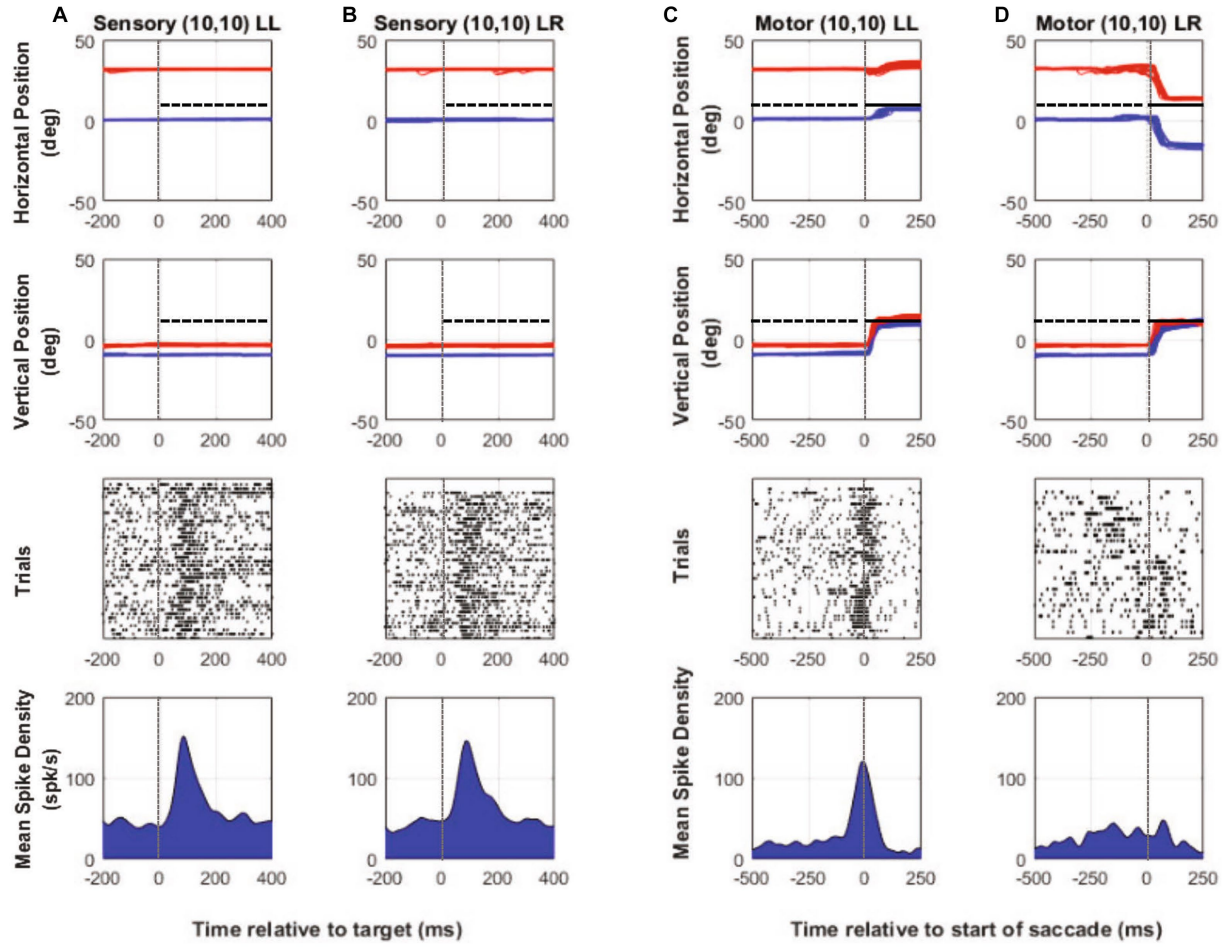
One-target testing example cell (M1). Target aligned eye and neural data (columns **A** and **B**) and saccade aligned eye and neural data (columns **C** and **D**) when the monkey used either the initially fixating left eye (columns **A,C**—LL trials) to acquire the eccentric target or the initially deviated right eye to acquire the same eccentric target on other trials (columns **B,D**—LR trials). In each column, top row is horizontal eye position (red—right eye, blue—left eye, and black—target), second row is vertical eye position, third row is raster showing incidence of neuronal spikes on each trial (each row in raster represents a single trial), and fourth row is the mean neuronal spike density function. Positive numbers on the position axes are rightward or upward positions and negative numbers are leftward or downward. For this example neuron, placing the target at (10, 10), which was also the neuron’s receptive location with respect to the initially fixating left eye, results in spontaneous fixation-switch on a subset of trials (columns **B,D**). As the sensory responses show in columns **(A)** and **(B)**, sorting the trials according to whether there would be an eventual fixation switch (column **B**) or not (column **A**) resulted in robust visual response for both but with a slightly smaller sensory peak in column **(B)** compared to column **(A)** (mean peak spike density sensory—LL peak: 151 spks/s, LR peak: 145.8 spks/s). Motor responses when the initially fixating left eye acquires the target (no-switch trials) is robust (column **C**) and is because the saccade is made to the neuron’s visual receptive field. On fixation-switch trials (column **D**), the motor response is absent because saccade that brings the previously deviated eye onto the target is in the opposite direction as the preferred saccade vector (mean peak spike density motor—LL peak: 120 spks/s, LR peak: 38.4 spks/s).

Our investigation of SC responses also led us to record from cells whose receptive fields lay in spatial locations where the monkey would use the same eye to acquire the eccentric target on some trials or would spontaneously switch fixation to acquire the target with the deviated eye on other trials. Thus with a singular placement of the target (one-target paradigm—Figure 1D), located in the purple shaded region of Figure 1E, we could compare fixation-switch with no-switch trials. One framework to consider the one-target paradigm is that there is a race between two neuronal populations; one population whose responses leads to generation of a saccade where the same eye acquires the target (no-switch) and another population where the previously deviated eye acquires the target (fixation-switch). In the case of the one-target paradigm, the two possible saccades are in opposite directions and therefore in different colliculi (Figure 1D). Recording from single SC neurons could potentially signal whether the neuron and by extension the neuronal population won the race or not. Raw data plots (Figures 6A–D) show neural and binocular eye data from an example cell during one-target testing. Figures 6A,B show visual responses on trials with no subsequent fixation-switch (Figure 6A) or with eventual fixation-switch (Figure 6B). The cell clearly shows a visual response in both conditions indicating that the neuron is responsive to placement of target in its receptive field irrespective of whether the subsequent saccade was generated or not. However, note that the peak visual response is reduced for the switch-trials compared to the no-switch trials. Since the saccades that led to no-switch (Figure 6C) are in opposite directions to those that led to fixation-switch (Figure 6D), we observed a clear difference in the motor firing as in a motor burst is observed in Figure 6C but there is no motor burst in Figure 6D.

**Figure 7:**
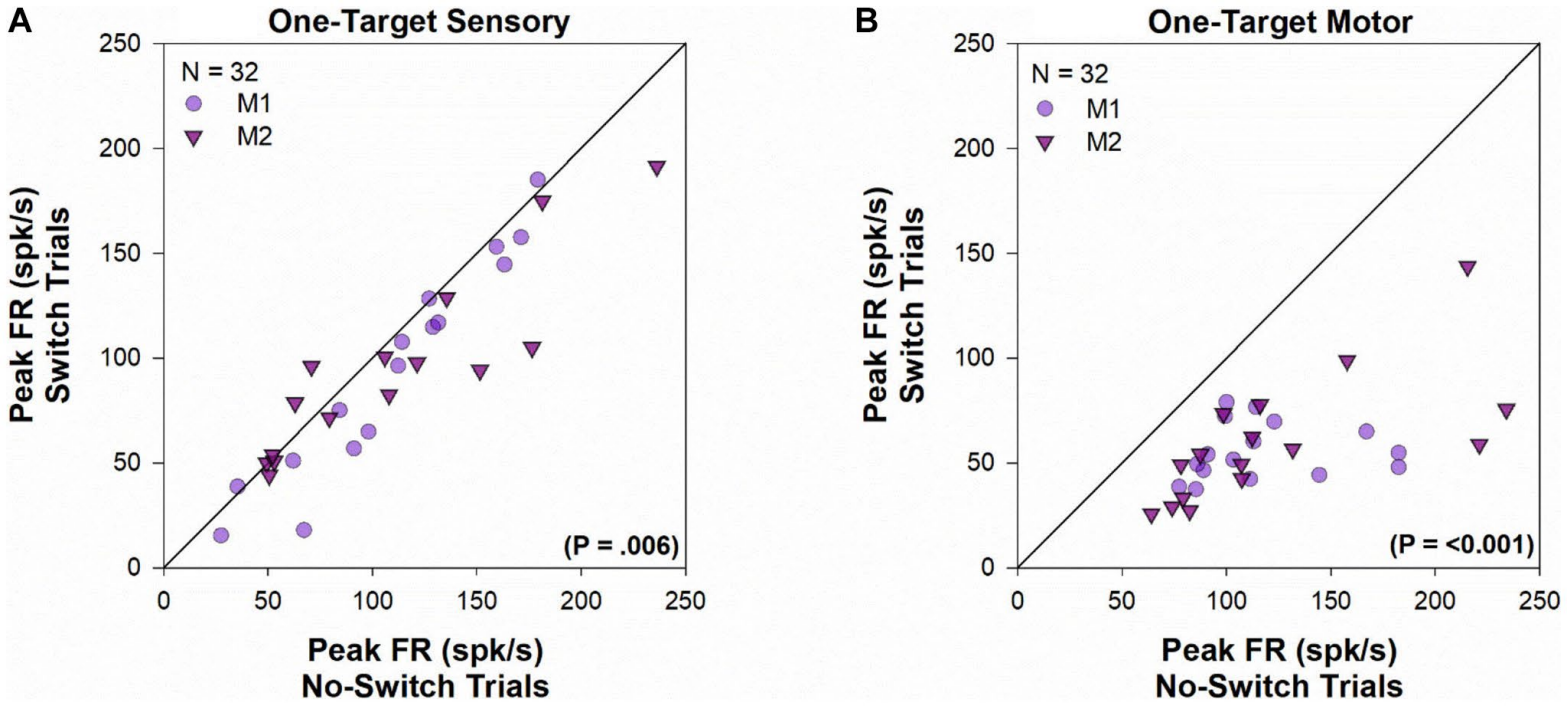
One-target visual and motor peak population response. Comparison of the peak visual response (panel **A**) or peak motor response (panel **B**) during fixation-switch trials and no-switch trials of cell population during one-target testing. On the *x*-axis is the mean peak response for trials in which the initially fixating eye acquired the target (no-switch trials) and on the *y*-axis is the mean peak response on trials in which the initially deviated eye acquires the target (fixation-switch trials). Each symbol (circles—M1; triangles—M2) represents one cell. Peak visual responses were defined as the maximal value of the average instantaneous frequency response occurring within 100 ms of target onset. Peak motor responses were defined as the maximal value of the average instantaneous frequency response occurring within 100 ms surrounding saccade onset. No-switch trials show reduced peak visual activity compared to fixation-switch trials indicating the effect of visual suppression. Peak motor activity in fixation-switch trials is diminished because the saccade vector is not in the preferred direction for the neuron.

The peak sensory and motor firing rate for the population of cells acquired in the one-target paradigm is shown in Figure 7. During the sensory period [*t*(31) = 2.951, *p* = 0.006, paired *t*-test], there was a significantly greater peak visual response when the fixating eye acquires the target (no-switch trials) when compared to trials where ultimately the deviated eye acquires the target (fixation-switch trials). The significant difference in sensory responses observed is consistent with the interpretation that trials with higher sensory firing led to winning the race and no-switch while lower firing on other trials led to subsequent fixation-switch wherein the oppositely directed saccade won the race to acquire the target. It is also consistent with the interpretation that the reduced sensory firing in some trials is evidence for dynamic visual suppression that is variable from trial to trial. Although we did not perform simultaneous recording in both colliculi, it is expected that for those trials where the deviated eye obtains the target, a population of cells in the other colliculus with appropriately located receptive fields are firing competitively to “win” to acquire the target. During the motor period (*Z* = −4.937, *p* ≤ 0.001, Wilcoxon Signed Rank Test), firing rate is significantly higher for no-switch trials since those are the only trials that result in a saccade that is in the optimal direction and amplitude of the neuron.

**Figure 8:**
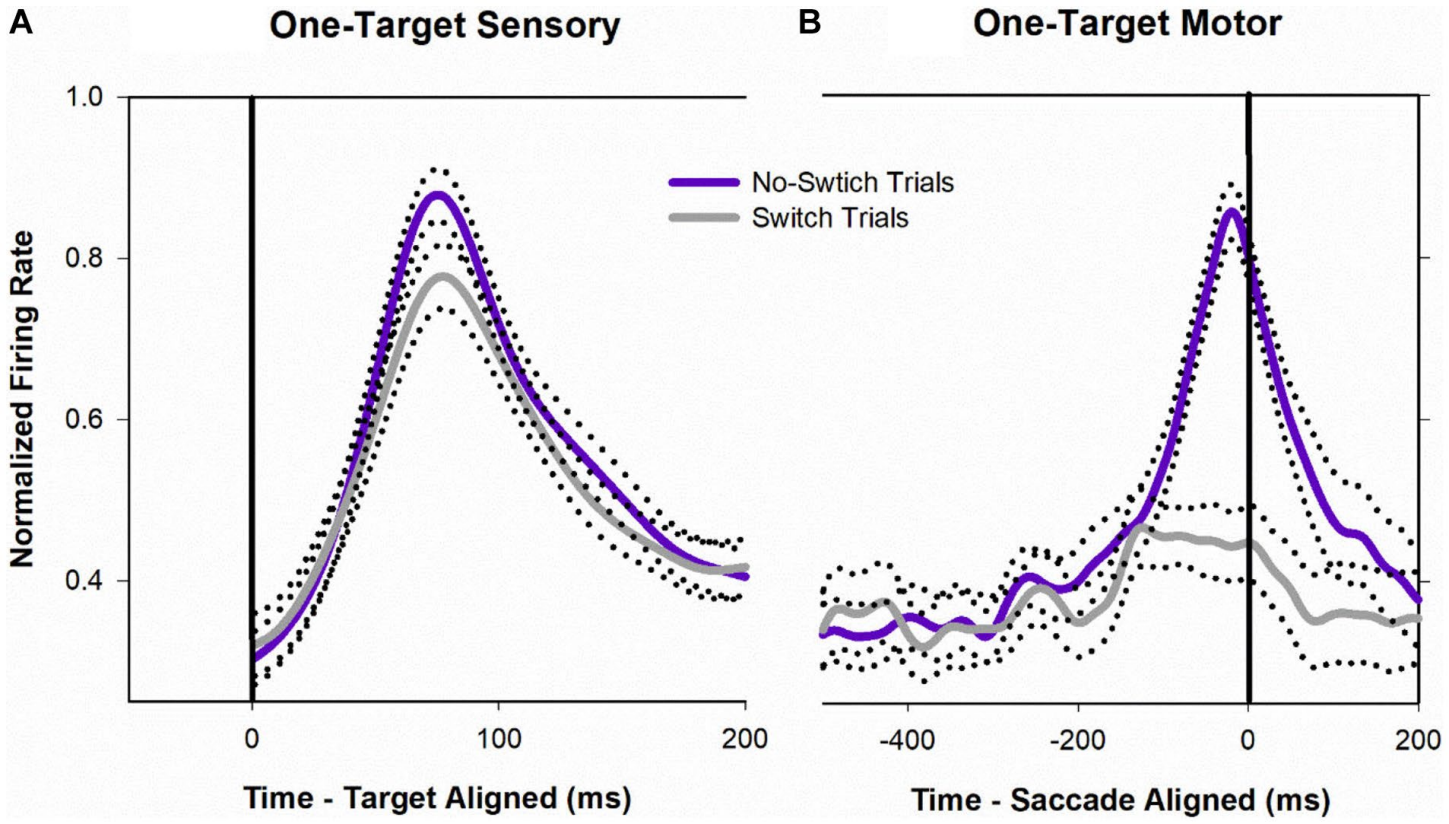
Normalized population (*n* = 32) activity of SC cells during one-target testing. Data are aligned at target onset (panel **A**; vertical line) or saccade onset (panel **B**; vertical line). Purple trace is mean activity when the initially fixating eye acquired the target (no-switch trials) and gray trace is mean activity when the initially deviated eye acquired the target (fixation-switch trials). Dotted traces indicate ±standard error of the mean (SE). The neuronal response of each cell was normalized based on its maximal response and then a population mean was obtained by averaging the individual cell responses. Note that because each contributing neuron was normalized individually and did not necessarily reach a peak at the same time as other neurons, the mean population activity does not reach a value of 1.0.

**Figure 9:**
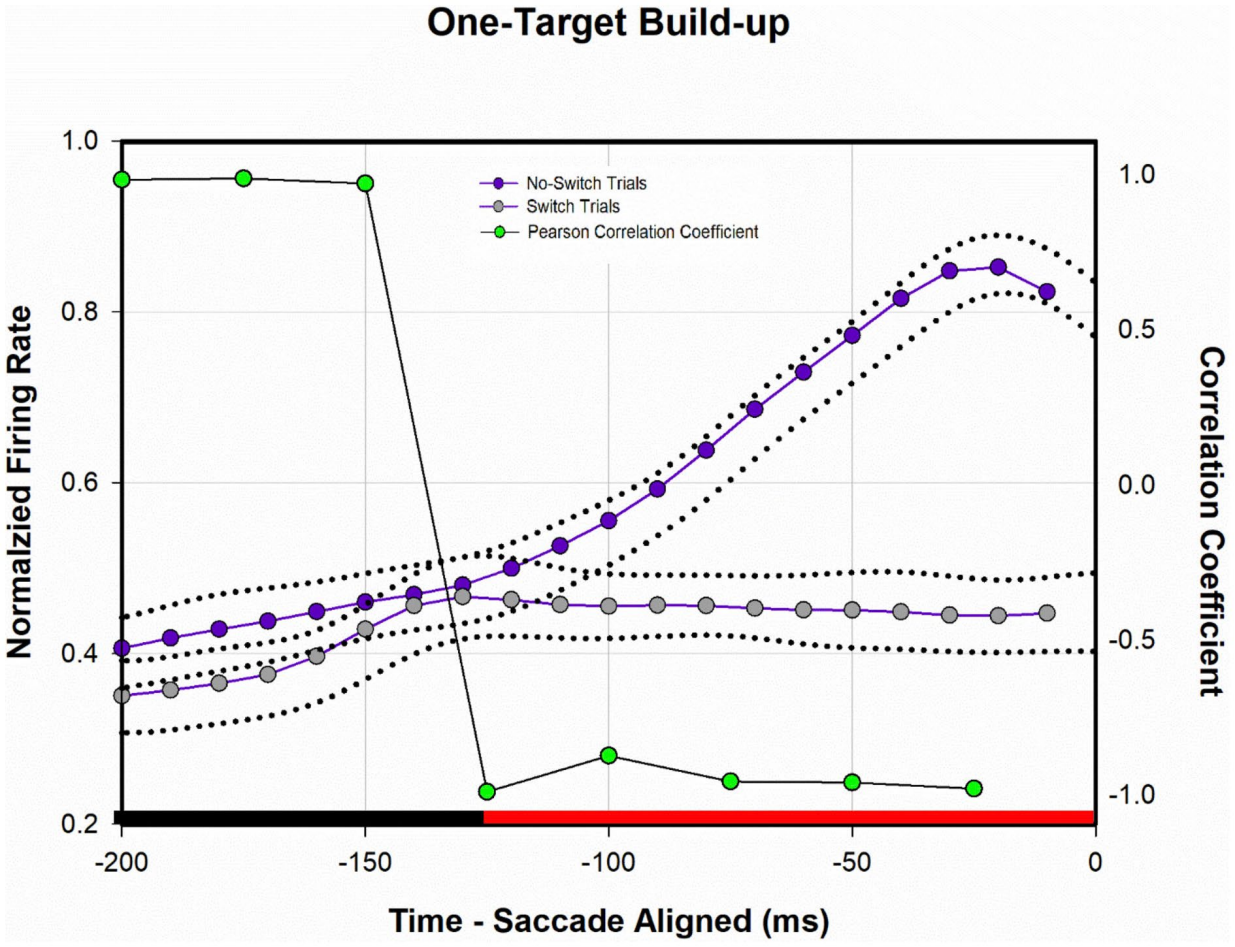
Analysis of normalized population activity during buildup period (*n* = 32) during one-target testing. The traces show the mean population neural response during one-target testing for the 200 ms period prior to saccade onset, which includes the buildup period. Dotted traces indicate ±SE. A Pearson correlation coefficient was developed with 25 ms bins of data between fixation-switch and no-switch trials. Black bars indicate a statistically significant positive correlation (Pearson Correlation) between fixation-switch (deviated eye acquires target) and no-switch (fixating eye acquires target) trials. Red bars indicate a statistically significant negative correlation (Pearson Correlation). The activity across the sample of buildup neurons during buildup period started to separate at around the 125 ms prior to saccade onset suggesting that this could be the time at which an eye choice is made.

Figure 8 shows a temporal profile of neuronal activity of the population during the one-target paradigm. Traces in purple are neuronal response during no-switch trials and traces in gray are neuronal response during fixation-switch trials. Clearly peak visual response is diminished on trials when a fixation-switch occurs (Figure 8A) and there is no subsequent motor burst (Figure 8B). To further understand potential competition between possible saccade vectors we investigated the firing rates during the buildup period which occurs 70–100 ms before the saccade is initiated as this could be the period that signals saccadic eye movement decisions (Munoz and Wurtz, 1995; Basso and Wurtz, 1998; Kim and Basso, 2008; Jun et al., 2021). The activity across the sample of neurons showed a reduction of buildup activity when the target was acquired by the deviated eye (Figure 8B). We performed a correlation analysis (Figure 9) within a 200 ms window before the saccade (which included the buildup period) between trials that resulted in fixation switch and those that did not. The 200 ms buildup period was divided into 25 ms bins to locate when the difference in buildup activity occurred and was furthermore categorized as an early buildup (first 100 ms) and a late buildup (last 100 ms) as has been done before by Basso and Wurtz (1998). The correlation analysis showed that there was a statistically significant negative correlation in buildup activity in the later buildup period (at ~125 ms) between fixation switch and no-fixation switch trials which putatively signals time of selection of the winning saccade.

## 4. Discussion

The mechanisms underlying selecting a single target from among two potential eccentric targets, within a binocularly aligned system, could hypothetically be also used in a strabismic monkey whose eyes are pointed in different directions and therefore must pick from two possible saccade vectors that would bring either the fixating or the previously deviated eye onto a single eccentric target. *The* SC is part of the target selection circuitry (Gandhi and Katnani, 2011) with a critical role assigned to the visuomotor buildup cells in identifying a “winner” (Glimcher and Sparks, 1992; Basso and Wurtz, 1998; Horwitz and Newsome, 2001). Among other evidence of target selection occurring during the buildup period, particularly relevant to our current work are studies that showed differences in buildup activity when saccades were made to targets in the receptive field compared to instances where saccades were made to distractors in the opposite field (Glimcher and Sparks, 1992; Thompson et al., 1996; McPeek and Keller, 2002; Jantz et al., 2013). For this target selection framework to be suitable for eye-choice behavior in strabismus, two elements must be proven true. First, retinal error information suitable to bring either eye onto the target via the subsequent saccade must be available at the same time. We found this to be true via the two-target paradigm. Second, the buildup activity and sensory activity should show clear indication of choosing from among the two targets. We found evidence supporting this second contention via the one-target paradigm.

### 4.1. Retinal error information is available from either eye in SC neurons

Behavioral studies of visual suppression and eye-choice behavior have shown a strong correlation between visual suppression of retinal areas and the eye that acquires the target via the saccade (Sireteanu, 1982; Das, 2009; Economides et al., 2012; Agaoglu et al., 2014). In exotropia, the nasal retina and parts of the temporal retina immediately close to the fovea are not suppressed in each eye (Economides et al., 2012). Consequently, a target appearing in unsuppressed retina of one eye or equivalently the suppressed retina of the fellow eye would be acquired by the unsuppressed eye. A prediction from these observations would be that perceptual visual suppression, wherever that might occur, would directly lead to eye choice and fixation switch behavior. While this is still largely true, our studies provide some more nuanced understanding and raises some new questions. The first observation is that, within the SC, visual or equivalently retinal error information associated with both the viewing and the deviated eye is available for subsequent oculomotor processing leading to eye-choice (two-target paradigm visual responses, Figure 4). This could be construed as an unexpected finding because the alternative prediction could have been that visual suppression in earlier visual areas results in the availability of SC visual responses that is *only* associated with the receptive field locations corresponding to the eye that would eventually acquire the target. The finding of visual information associated with each eye supports a framework of competition and places the SC within that computational circuit. Note that our finding agrees with those of Economides et al. (2018) who used a sparse noise stimulus during fixation to investigate receptive field locations with respect to viewing and deviated eyes in surgically induced strabismic monkeys.

Although retinal error information associated with both eyes is indeed available within SC of strabismic monkeys, the amplitude of visual responses was marginally (statistically significant) higher when targets were presented in the receptive field of the eye that would eventually acquire the target (Figure 3). We suggest that this may be evidence for small interocular suppression that potentially biases the SC competition toward choosing one eye over the other to acquire the target. It is possible that the small statistical difference in visual responses is because the deviated eye position tends to be more unstable compared to that of the fixating eye and therefore the LED positions do not fall on the same location within the receptive field location of each eye. We attempted to account for this possible confound by only selecting trials with matching saccade amplitudes in the two conditions but cannot discount possible small effects due to slight shifts in LED presentation within the receptive field. In any case, although small interocular suppression may be present for sensory information, it does not influence the final saccade that is generated.

### 4.2. Sensory suppression, buildup activity and eye-choice behavior

The second major finding supporting a competition framework for eye-choice behavior in strabismus are the analysis of sensory and buildup activity with the SC visuomotor neurons, within the one-target paradigm. Since the two possible saccade vectors in the one-target paradigm are in opposite directions, direct proof of competition between the two colliculi might have been had by simultaneous recording from both colliculi and directly comparing neuronal activity of pairs of neurons (one from each colliculus) that corresponded to the two possible saccade outcomes (Figure 1D—V3 vs. V4). We were unfortunately not able to perform this challenging dual-recording experiment made further complicated by needing to find matching cells whose saccade vectors exactly corresponded to the two possible outcomes of eye choice to the 1-target presentation. However, in its absence, we compared the sensory and buildup activity of the same neuron in trials where the saccade vector corresponded to the neuron’s preferred direction to those in which it did not, i.e., other eye acquired the target. We found evidence of sensory suppression (Figures 7, 8) on trials that led to fixation-switch in that trials that led to fixation switch showed reduced peak visual responses. We believe that it is unlikely that the reduced firing rate for fixation switch trials is an eye position related gain modulation (eye position gain fields) that is coming about because the deviated eye is at a different position in the orbit. The reason we consider this explanation unlikely is that our population contained a mix of cells recorded in the right and left colliculus and also was a mix of cells with initial right eye or left eye fixation. Interestingly, the difference in visual response peaks between fixation-switch trials and no-switch trials is only about 15% in magnitude. The question that is raised via this finding is whether these small differences are the extent of visual suppression related neural responses that should be expected in other visual areas and whether such small differences in visual responses is sufficient to prevent perceptual diplopia. Recently, Economides et al. (2021) reported the absence of visual suppression within V1 neurons suggesting that a neural substrate for visual suppression is in higher order visual cortical areas and it would be interesting to compare magnitudes of neuronal visual suppression in those visual cortical areas with what we observe in SC (Das, 2009; Economides et al., 2012, 2014; Agaoglu et al., 2014, 2015; Ramachandran and Das, 2020). The source of apparent visual suppression in SC is yet not clear but it could either be directly from higher order visual cortical areas noted above or indirectly via projections from LIP or FEF, structures with which the SC shares a target selection responsibility.

We also found (Figure 9) that at approximately 125 ms before the saccade there was a dropoff in buildup activity for trials where the saccade vector (and therefore the eye that is chosen) did not correspond to the preferred direction for the neuron. The drop off in build-up activity was predictive of absence of subsequent motor burst and therefore generation of the saccade. This type of buildup response is similar to that observed in a variety of target selection type studies in normal monkeys (Hanes and Schall, 1996; Thompson et al., 1996; Basso and Wurtz, 1998; McPeek and Keller, 2002) and provides confidence in a competition framework for eye choice behavior in strabismus. The prediction here is that an “anti-neuron” in the other colliculus would show the opposite sequence of activity, i.e., increase in buildup when an appropriate saccade is chosen, with a similar time course.

### 4.3. Study limitations and future work

Although typical of studies involving non-human primate physiology, a general limitation with such studies is the limited numbers of animals (*n* = 2 in our study). Another limitation is that we did not explicitly test the depth of visual suppression in these animals. Although not impossible, this parameter would be difficult to measure in non-human primates. We have chosen instead to base our expectation on studies by Economides et al. (2012) where they psychophysically tested visual suppression in humans with strabismus and showed that suppression patterns matched spatial fixations patterns that we found. In target selection in normals, many parts of the brain have access to visual error signals including but not limited to the FEF, LIP, and SC (Hanes and Schall, 1996; Schall and Thompson, 1999; Bisley and Goldberg, 2010). Because of the overlap in the target selection and saccadic circuit it would be useful to utilize sophisticated target selection frameworks developed via studies in normal animals to understand how the signals transforming sensory information to motor behavior incorporating eye choice is developed in other parts of the brain such as the FEF and LIP in strabismic monkeys (Camalier et al., 2007; Murthy et al., 2009). There remains a possibility that the firing patterns observed in the SC are simply reflective of decisions already made in other areas such as the FEF or eye specific priority maps developed in other areas such as the LIP.

## Data availability statement

Analyzed summary data included in manuscript will be made available upon request. Requests to access the datasets should be directed to vdas@central.uh.edu.

## Ethics statement

The animal study was approved by University of Houston Institutional Animal Care and Use Committee. The study was conducted in accordance with the local legislation and institutional requirements.

## Author contributions

SR: Conceptualization, Data curation, Formal analysis, Investigation, Methodology, Software, Validation, Visualization, Writing – original draft, Writing – review & editing. VD: Conceptualization, Funding acquisition, Methodology, Project administration, Resources, Software, Supervision, Writing – review & editing.
